# Incorporating oral health care education in undergraduate nursing curricula - a systematic review

**DOI:** 10.1186/s12912-020-00454-6

**Published:** 2020-07-14

**Authors:** Vandana Bhagat, Ha Hoang, Leonard A. Crocombe, Lynette R. Goldberg

**Affiliations:** 1 Centre for Rural Health (CRH), E Block, Newnham Campus, University of Tasmania (UTas), Launceston, Australia; 2 CRH, E block, Newnham Campus, UTas, Launceston, Australia; 3 CRH, ABC Building, 1 Liverpool Street, Hobart, Hobart CBD Campuses, UTas, Hobart, Australia; 4 Wicking Dementia Research & Education Centre, Room 421C (Level 4), Medical Science 1, UTas, Hobart, Australia

**Keywords:** Oral health, Oral care, Older people, Nursing, Attitudes, Education, Understanding, Knowledge, Curricula, Interprofessional

## Abstract

**Background:**

The recognised relationship between oral health and general health, the rapidly increasing older population worldwide, and changes in the type of oral health care older people require have raised concerns for policymakers and health professionals. Nurses play a leading role in holistic and interprofessional care that supports health and ageing. It is essential to understand their preparation for providing oral health care.

*Objective:* To synthesise the evidence on nursing students’ attitudes towards, and knowledge of, oral healthcare, with a view to determining whether oral health education should be incorporated in nursing education.

**Methods:**

*Data sources*: Three electronic databases - PubMed, Scopus, and CINAHL.

*Study eligibility criteria, participants and interventions:* Original studies addressing the research objective, written in English, published between 2008 and 2019, including students and educators in undergraduate nursing programs as participants, and conducted in Organisation of Economic Co-operation and Development countries.

*Study appraisal and synthesis methods:* Data extracted from identified studies were thematically analysed, and quality assessment was done using the Mixed Methods Appraisal Tool.

**Results:**

From a pool of 567 articles, 11 met the eligibility criteria. Findings documented five important themes: 1.) nursing students’ limited oral health knowledge; 2.) their varying attitudes towards providing oral health care; 3.) the need for further oral health education in nursing curricula; 4.) available learning resources to promote oral health; and 5.) the value of an interprofessional education approach to promote oral health care in nursing programs.

*Limitations:* The identified studies recruited small samples, used self-report questionnaires and were conducted primarily in the United States.

**Conclusions:**

The adoption of an interprofessional education approach with a focus on providing effective oral health care, particularly for older people, needs to be integrated into regular nursing education, and practice. This may increase the interest and skills of nursing students in providing oral health care. However, more rigorous studies are required to confirm this. Nursing graduates skilled in providing oral health care and interprofessional practice have the potential to improve the oral and general health of older people.

## Introduction

### Rationale

Oral health is measured by the absence of orofacial pain, oral infection, periodontal (gum) diseases, tooth decay, tooth loss, and other orofacial diseases and disorders that can affect a person’s overall physical and mental health, and social well-being [1–3]. This is a particular concern for older people [2–5]. The world’s population is ageing rapidly [4]. Living longer brings challenges when meeting the complex healthcare needs of many older people and ensuring their quality of life. Currently, there is a profound disparity in the oral health of the older population, even in high-income countries [5].

Worldwide, the oral health of older people, defined as those over 65 years of age, is poor with a high prevalence of dental caries, periodontal diseases, dry mouth problems, and incremental tooth loss [5, 6]. Oral health problems often lead to malnutrition, and difficulty with speech and swallowing [7]. There is increasing evidence of the association of periodontal problems with systemic conditions including type II diabetes, osteoporosis, cardiovascular problems such as myocardial infarction, stroke, coronary heart disease, and aspiration pneumonia that may lead to unplanned hospitalisations [8–10]. Poor oral health impacts morbidity, mortality, and recovery time after treatment [11, 12]. Pain and suffering resulting from oral health problems may influence older people’s mood and behaviour, particularly if they have difficulty in communicating their discomfort [13]. Poor dental appearance and bad breath can lower self-esteem and exacerbate social isolation [14]. Thus, oral health problems can have profound physical, psychological, social, and economic consequences.

Most oral health problems experienced by older people are preventable or treatable [15]. However, they remain underdiagnosed and untreated due to the lack of effective, efficient, and equitable distribution of oral health services [15]. Reasons for the inadequate delivery of oral health services to older people include limited resources, poor understanding of oral care among nursing staff, lack of interprofessional collaboration, and inadequate policy protocols [16, 17]. The lack of time, competing priorities, a high workload, and staffing issues are also significant barriers for providing oral care to older people [18].

The provision of quality and timely oral health care services to the rapidly increasing older population has become a large challenge for policymakers and health professionals [9, 19, 20]. Many changes have occurred in the oral health care needs of the older population in the twenty-first century due to the preservation of natural teeth, and the placement of complex prostheses such as crowns, bridges, overdentures, and implants. These changes highlight the need for staff trained in providing oral health care to older people [21, 22]. With increasing age and ill-health, many people need assistance with their oral and general health care [23, 24]. This is particularly true for dependent older adults in residential care communities and hospitals. However, oral health care is a low priority for non-dental health professionals [6, 25–27].

Interprofessional education and collaborative practice have been recognised as a valuable approach to alleviate the global health workforce crisis and prepare a health workforce that will better respond to local health needs and ensure safe, holistic practice [28]. The World Dental Federation (FDI) also supports the need for interprofessional education and collaborative practice to improve access to oral health services [29]. Involving nurses, primary health care workers, and other allied health professionals in oral health care, will increase the national capacity to reach vulnerable and underserved population groups, including older people [6]. Nurses account for a large proportion of the health care workforce and are often present at the point of care or supervising direct caregivers [30, 31]. Therefore, oral health care education and training are essential for graduating nurses to improve the oral and systemic health of older people [32–36]. Such education and practice provided with an interprofessional approach enables nursing students to contribute, learn and work effectively with other professionals involved in oral health [29].

Nurses provide care to older people in various settings such as hospitals, residential aged care, rehabilitation units, as well as in the community. Community nurses can educate and empower older people to take an active role in their oral care to prevent oral problems [37]. Nurses working in residential communities can take a leadership role in ensuring oral health care is integrated into routine nursing care [38]. Nurses can screen each resident’s oral health upon admission, assess the need for an examination by a dental professional, and prepare and monitor an oral health care plan [25, 39, 40]. Registered nurses can train and supervise personal care assistants in providing support to residents to maintain oral hygiene, monitor adequate nutrition, and identify signs of oral diseases [33]. Similarly, in hospitals, nurses can promote oral health, screen for any suspicious oral pathology, and make appropriate referrals [41]. Given interprofessional support, nurses can improve and maintain the oral health of older people when immediate access to an oral health therapist is not available [23].

### Objective

To synthesise the evidence on nursing students’ attitudes towards, and knowledge of, oral health care, with a view to determining whether oral health education should be incorporated in nursing education. To our best knowledge, no previous study has summarised the literature on this topic.

### Research questions

1. What do nursing students understand about oral health care?
2. What are the attitudes of nursing students towards providing oral health care?
3. Is there evidence of oral health education and training in nursing curricula?

## Methods

A systematic review was performed following Preferred Reporting Items for Systematic Reviews and Meta-Analyses (PRISMA) guidelines [42].

### Eligibility criteria

Original studies addressing the research questions, written in English, published between 2008 and 2019, including students and educators in undergraduate nursing programs as participants, and conducted in Organisation of Economic Co-operation and Development (OECD) countries. The review excluded studies involving students from certificate nursing courses, graduate nursing programs, and midwives. Studies reported in conference proceedings, short communications, thesis, or book chapters were also excluded.

### Information sources and search

**Fig. 1:**
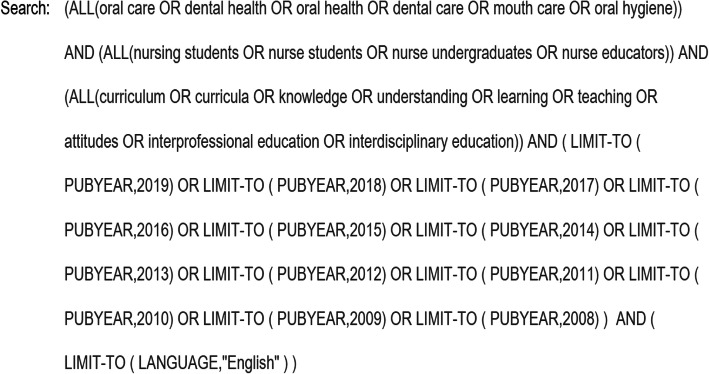
Full electronic search strategy

Three electronic databases were searched: PubMed, Scopus, and CINAHL. Boolean operators with the following keywords and strategy were used: (oral care OR dental health OR oral health OR dental care OR mouth care OR oral hygiene) AND (nursing students OR nurse students OR nurse undergraduates OR nurse educators) AND (curriculum OR curricula OR knowledge OR understanding OR learning OR teaching OR attitudes OR interprofessional education OR interdisciplinary education). A detailed example of the search strategy used for Scopus is outlined (Fig. 1). This search strategy was adapted for each of the databases.

### Study selection

All original studies, including quantitative, qualitative, and mixed-method studies, were selected if they met the eligibility criteria. After an initial search and removing duplicates from the search list, titles and abstracts were independently screened by two authors (VB, HH) and the full texts of identified papers were then sought. Studies not fitting the eligibility criteria were excluded before the full text was reviewed. In cases of disagreement, two additional authors (LC, LG) were consulted to resolve any conflicts. Disagreements were resolved with consensus by referring back to the protocol. Research data were synthesised systematically, and the quality of the included studies was then evaluated.

### Data extraction

The data collection form was developed by two authors (VB, HH) referring to previous systematic reviews in the related field; data extraction was then performed independently by VB. The accuracy of the extracted data was verified by the second author (HH). Information collected from the identified articles for this systematic review included: country and setting, details of participants, objectives of the study, research design, description of the main findings related to the three research questions, and the reported limitations of each study (Table 1).

**Table 1:** Characteristics of identified studies

Reference	Setting and country	Objective	Participants	Research Design	Main findings	Limitations
Clemmens, Rodriguez & Leef, 2012 [43]	A large private urban university, USA	To identify the knowledge, attitudes, and practices of baccalaureate nursing students regarding oral health assessment.	A convenience sample of 163 baccalaureates nursing students	Descriptive cross-sectional survey	Although nursing students felt that oral health is an essential component in nursing practice, they lacked a complete understanding of the critical components of an oral health examination and promotion. Almost all the participants (97%) believed that they had a good understanding of oral health assessment, but only 25% were able to recognise the components of oral health assessment. Less than 2% reported performing an oral health assessment for every patient.	The validity and reliability of the survey instrument were not established on the cohort of nurses. Moreover, data collected from a single university so results cannot be generalised.
Doğan, 2013 [44]	Marama University, Turkey	To assess the differences in oral health behaviour and attitudes between nursing and dental students.	157 nursing students and 71 dental students	A comparative descriptive cross-sectional survey	The attitudes of dental students to oral health were significantly more positive than that of nursing students (*p* < 0.001). The proportion of students avoiding visiting a dentist until they had a painful oral condition was significantly higher among nursing students than dental students. A third (33%) of nursing students in comparison to 6% of dental students thought that they could not avoid having false teeth in old age. The dentist found that dental students (58%) were better at brushing in comparison to nursing students (25%). The variation in attitudes and behaviour of nursing and dental students reflected the significance of practical training and curriculum.	Data collected from students of one university only so results cannot be generalised.
Haresaku et al., 2018 [45]	Nursing, dental, and dental hygiene school belonged to school cooperation in Fukuoka Prefecture, Japan	To identify the weak points in knowledge, attitudes, and factors building positive willingness to practice oral health care among nursing and oral healthcare students.	First-year nursing (119), dental (88), and dental hygiene students (64)	Analytical cross-sectional survey	Nursing students had the poorest knowledge and attitudes towards oral health care compared to other student groups. About 40% of nursing students felt they did not know much about oral healthcare, and 39.2% were not interested in oral healthcare. Only half of all students knew that oral health care is necessary to prevent general health problems like cardiovascular disease and aspiration pneumonia. Acknowledging and building interest among nursing students about oral healthcare is associated with a positive willingness for oral health practices. Therefore, it is essential to develop a collaborative nursing oral health curriculum to motivate nursing students.	Results collected from a single nursing school, so the attitudes and behaviours cannot be generalised.
Pai, Ribot, Tane, & Murray, 2016 [31]	Four Charles Sturt University campuses in regional NSW, Australia	To assess final year nursing students’ awareness of the periodontal disease.	30 final year nursing students	Cross-sectional quantitative study	Nursing students were unable to determine the causes of periodontal disease, but their general knowledge was adequate regarding issues related to periodontal disease. Most participants indicated a lack of confidence in oral health care practice and recommended including more oral health content in the nursing curriculum.	A small sample size and data were collected from campuses belonging to the same university, so results cannot be generalised.
Grant et al., 2011 [46]	George Brown College (GBC) dental clinic, Ontario, Canada	To report the lessons learned from the Interprofessional education (IPE) initiative between dental hygiene and BScN students and identify future directions.	Eight 2nd year Dental Hygiene (DH) students participated in teaching oral health to 200 1st year Bachelor of Science (BScN) nursing students and, 15 2nd year BScN students participated in teaching blood pressure measurement to each of 4 pairs of DH students.	Quasi-experimental post-survey pilot study	Both student groups enjoyed working with each other and sharing skill sets. Students experienced each other’s professional language, which is an essential step for good communication between health professionals. DH students found that BScN students did not have an adequate understanding of the theory of oral health assessment or daily oral care.	A pilot study from a single site so results cannot be generalised.
Czarnecki, Kloostra, Boynton, & Inglehart, 2014 [47]	Pediatric dentistry clinic, USA	To evaluate interprofessional education among nursing and dental students, and pediatric dentistry residents.	Experimental group: Data collected from 33 1st year nursing students, 40 3rd year dental students, and six pediatric dentistry residents. Control group: Data collected from 1st and 2nd-year dental students at the beginning and end of the term.	Quasi-experimental pre- and post-test survey	Nursing students showed significant improvement (*p* < 0.05–0.001) in their oral health behaviour, knowledge, and attitudes regarding the importance of oral care and translating theory into nursing practice. Dental students also improved their attitudes toward the importance of nurses’ engagement in oral health assessment and promotion. All students agreed that interprofessional clinical placements are a better way of learning than only lectures.	Results based on a sample from one university only. No control group among nursing students.
Farokhi, Muck, Lozano-Pineda, Boone, & Worabo, 2018 [48]	Church-based clinic run in partnership with San Antonio Refugee Health Clinic and University of Texas Health San Antonio, USA	To assess the oral health literacy and knowledge gained by patients, community members, medical and nursing students after participating in an IPE activity.	Convenience sampling of nursing students (34), medical students (38), community members/parish (17), refugee patients (151)	Quasi-experimental pre- and post-survey	Ten dental, two dental hygiene, ten medical, and ten nursing students operated the clinic every week. Pre- and post-survey scores (*p* < 0.0001) showed that IPE benefitted all participants as measured by increased oral health literacy scores among all groups. The program provided patient management in a supportive team culture by expanding their learning of oral-systemic disease connections.	Convenience sampling from a single area.
Lewis, Edwards, Whiting, & Donnelly, 2018 [49]	University and vocational education sectors, Australia	To test if oral health resources designed for workforce training were relevant to entry-level nursing or age care qualifications.	Bachelor of Nursing (*n* = 41), Diploma of Nursing (*n* = 66) and Certificate in Aged Care course students (*n* = 17) and educators (6); two educators from each course	Mixed-method study	This study validated Building Better Oral Health Communities resources as an effective learning and teaching package for entry-level nursing and age care qualifications. Students and educators were highly satisfied with the study materials in promoting interest and providing insight into a comprehensive approach to oral health care. Students’ learning outcomes showed consistently positive attitudes and enhancements in oral health knowledge and skills.	Small sample size and non- random sampling. Results are based on self-reporting rather than a clinical assessment of oral health competency.
Nierenberg et al., 2018 [50]	School gymnasium, Appalachia, USA	To assess dental and nursing students’ reflections on an inter-professional service-learning experience in Appalachia.	36 dental and nursing students from University at Buffalo, NY, USA. Of 31 participants who completed the demographic questions, 21 dental students were in the third year, and ten baccalaureate nursing students were seniors (4th year).	Cross-sectional qualitative study	Dental and nursing students’ exposure to rural patients who often lack dental care and have severe oral health problems impacted ‘their’ appreciation of interprofessional practice and their willingness to provide care in underserved settings. IPE facilitated care through teamwork, with students gaining mutual respect, confidence, and an increased understanding of the relationship between oral and overall health. Underserved communities benefit tremendously from interprofessional clinical practice as they can consult with multiple providers at one place on the same day.	No control group and results collected from one single site.
Coan et al., 2019[51]	Two local hospitals with the dedicated educational unit, Indiana, USA	To implement and evaluate a collaborative event with patients to help develop dental hygiene and nursing students inter-professional competence	24 dental hygiene and 25 nursing students at the University of Southern Indiana	Retrospective pre-post survey design	15 out of 24 dental hygiene and all 25 nursing students completed the Interprofessional Collaborative Competency Attainment Survey (ICCAS). Results showed significant improvement from pretest to post-test for nursing students (85% of items on ICCAS with *p* ≤ 0.004–0.0001) and dental hygiene students (75% of items on ICCAS). Therefore, structured interprofessional collaborative practice in hospital settings showed a positive effect in developing interprofessional competencies among nursing and dental hygiene students. The collaborative practice helped students from both professions to consider patients’ oral health needs and implications of improved oral health for patients’ overall systemic health.	Small sample size and retrospective survey, which causes the risk of recall bias.
Dsouza et al., 2019 [52]	University of North Carolina at Chapel Hill, USA	To evaluate the influence of an educational intervention on knowledge, confidence, practice behaviours, and perceived barriers of nursing students regarding preventive oral health services.	64 first-year Accelerated Bachelor of Nursing students	Quasi-experimental pre-post survey design	Pre-survey results indicated that 77% (*n* = 33) of first-semester accelerated Bachelor of Nursing students had a poor or very poor level of oral health education. Only 7% of students reported that they were providing oral counselling and referrals before the intervention. The post-survey questions showed improvement in oral health knowledge scores and confidence in oral screening and counselling. A significant increase was noticed in the willingness to implement oral health services during clinical visits (*p* < 0.0001). Post survey qualitative data also documented the benefits of hands-on learning experiences with oral screening, counselling, fluoride varnish application, and referrals. Students found learning from dental hygiene educators useful.	Small sample size from a single nursing school and the absence of a control group

### Data synthesis

Extracted data were analysed thematically to produce a narrative description of the findings. While thematic synthesis is commonly used for qualitative research outcomes, it can be used for quantitative research outcomes when there is heterogeneity in measurements. Therefore, the process of thematic synthesis was chosen to narrate the findings of this review [53]. The thematic analysis was conducted according to Braun and Clarke’s guidelines: familiarisation with the data, coding, developing potential themes, reviewing themes, defining themes and reporting data relating to research questions [54]. The coding process involved segmenting data into similar groups and identifying the relationship between codes. After finishing the coding process, codes were grouped into descriptive themes that captured similarities in the data across identified studies. Finally, selected themes were reviewed, and synthesised data were finalised in relation to the research questions.

A meta-analyses of the identified studies was not possible because of the small number of studies, participants, and heterogeneity.

### Quality assessment

The quality assessment of the identified studies was done by two authors (VB, HH) using the Mixed Methods Appraisal Tool (MMAT) [55]. All studies were screened regarding the clarity of their research questions, and whether collected data addressed the research questions. Studies that passed the screening were then appraised using methodological quality assessment questions relative to the study design. Studies that met all assessment criteria scored 1; studies that met fewer criteria scored less than 1.

## Results

### Study selection

**Fig. 2:**
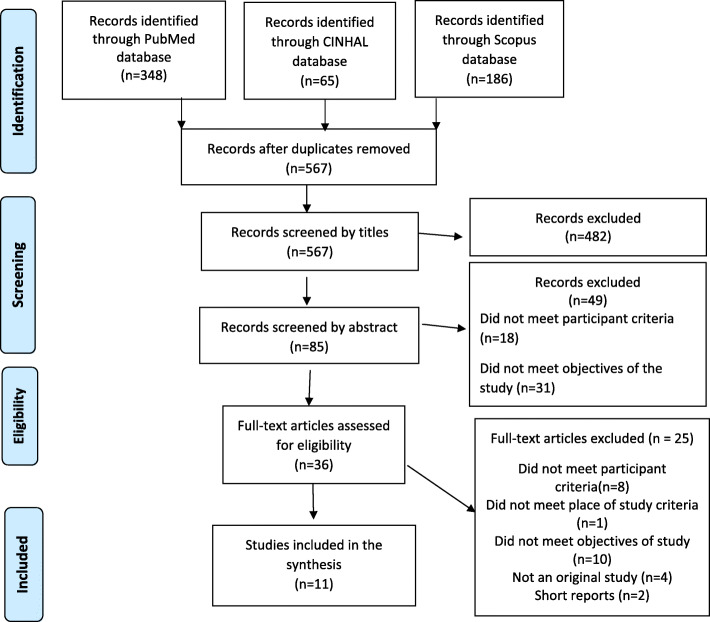
PRISMA flowchart detailing search results and the selection of studies

From a pool of 567 articles, 11 met the eligibility criteria (Fig. 2). A large number of articles were excluded based on the wording of their titles (482), 49 were excluded from reading the abstract, and a further 25 were excluded after reading the full text. Finally, 11 studies were included in this paper. Of the 11 studies, six were conducted in the United States, two in Australia, and one each in Japan, Turkey, and Canada. Studies evaluating nursing students’ oral health knowledge and attitudes of oral health care used cross-sectional survey design; intervention studies assessing the impact of the inclusion of oral health components in nursing curricula used quasi-experimental pre-post survey design, post-survey design, retrospective pre-post survey design, and cross-sectional qualitative design. The study evaluating oral health care resources for older people used a mixed-method design.

The main findings of the 11 identified studies were 1.) nursing students’ limited oral health knowledge; 2.) their varying attitudes towards providing oral health care; 3.) the need for further oral health education in nursing curricula; 4.) available learning resources to promote oral health; and 5.) the value of an interprofessional education (IPE) approach to promote oral health care in nursing programs. The results of the quality assessment of the identified papers are shown in Tables 2, 3, 4 and 5.

**Table 2:** Quantitative descriptive critical review analysis of four identified studies

Critical Appraisal Checklist Quantitative Descriptive	Clemmens et al., 2012[43]	Dogan, 2013 [44]	Haresaku et al., 2018 [45]	Pai et al., 2016 [31]
Screening question (SQ)1. Are there clear research questions?	Yes	Yes	Yes	Yes
SQ 2. Does the collected data address the research questions?	Yes	Yes	Yes	Yes
1. Is the sampling strategy relevant to address the research question(s)?	Yes	Yes	Yes	Yes
2. Is the sample representative of the target population?	No	No	No	No
3. Are the measurements appropriate (clear origin or validity is known or standard instrument)?	No	Yes	Yes	Unclear
4. Is the risk of nonresponse bias low?	Yes	Yes	Yes	No
5. Is the statistical analysis appropriate to answer the research question(s)?	Yes	Yes	Yes	Yes
Overall quality score*	0.6	0.8	0.8	0.4

*Overall quality score: Studies met all assessment criteria scored one and studies met fewer criteria scored less than one

**Table 3:** Quantitative critical review analysis of four identified studies

Critical Appraisal Checklist Quantitative non-randomised study	Czarnecki et al., 2014 [47]	Farokhi et al., 2018 [48]	Dsouza et al., 2019 [52]	Coan et al., 2019 [51]	Grant et al., 2011 [46]
SQ1.Are there clear research questions?	Yes	Yes	Yes	Yes	No
SQ2.Do the collected data allow one to address the research questions?	Yes	Yes	Yes	Yes	-**
1. Are the participants representative of the target population?	Yes	No	Yes	No	-**
2. Are measurements appropriate regarding both the outcome and intervention (or exposure)?	Yes	Yes	Yes	Yes	-**
3. Are there complete outcome data?	Yes	Yes	Yes	Yes	-**
4. Are confounders accounted for in the design and analysis?	Yes	No	Yes	Yes	-**
5. During the study period, is the intervention administered (or exposure occurred) as intended?	Yes	Yes	Yes	Yes	-**
Overall quality score*	1.0	0.6	1.0	0.8	-**

*Overall quality score: Studies met all assessment criteria scored one and studies met fewer criteria scored less than one

**According to MMAT, further appraisal may not be feasible or appropriate when answer is “‘No’ or ‘Cannot ‘tell’ to screening question 1 or 2

**Table 4:** Qualitative critical review analysis of Nierenberg et al., 2018 [50]

Critical appraisal checklist Qualitative study	
SQ 1. Are there clear research questions?	Yes
SQ 2. Do the collected data allow one to address the research questions?	Yes
1. Is the qualitative approach appropriate to answer the research question(s)?	Yes
2.Are the qualitative data collection methods adequate to address the research question(s)?	Yes
3.Are the findings adequately derived from the data?	Yes
4.Is the interpretation of results sufficiently substantiated by data?	Yes
5. Is there coherence between qualitative data sources, collection, analysis and interpretation?	Yes
Overall quality score*	1.0

*Overall quality score: Studies met all assessment criteria scored one and studies met fewer criteria scored less than one

**Table 5:** Mixed method critical review analysis of Lewis et al., 2018 [49]

Critical appraisal checklist Mixed method study	
SQ1. Are there clear research questions?	Yes
SQ2. Do the collected data allow one to address the research questions?	Yes
1. Is there an adequate rationale for using a mixed-method design to address the research questions?	Yes
2. Are the different components of the study effectively integrated to answer the research question(s)?	Yes
3. Are the outputs of the integration of qualitative and quantitative components adequately interpreted?	Yes
4. Are divergences and inconsistencies between qualitative and quantitative results adequately addressed?	Yes
5. Do the different components of the study adhere to the quality criteria of each tradition of the methods involved?	Yes
Overall quality score*	1.0

*Overall quality score: Studies met all assessment criteria scored one and studies met fewer criteria scored less than one

### Synthesis of results: five identified themes

#### Limited knowledge of oral health care among nursing students

Only three studies [31, 43, 45] assessed the oral health knowledge of nursing students. Using convenience samples ranging from 30 to163 students, each study used different questionnaires. Data were collected from students belonging to a single university or at different campuses of the same university. Studies conducted in the US and Japan showed students had limited oral health care knowledge and inadequate understanding of the crucial elements of an oral health assessment and promotion of effective oral health practices [43, 45]. Only 25% of all participants in the US-based study by Clemmens et al. [43] were able to recognise the critical components of oral health assessment, despite a majority of students thinking they understood these components. In Japan, Haresaku et al. [45] found that only half of the nursing students knew that oral health diseases could have an impact on systemic health. An earlier study by Pai et al. [31] in Australia showed nursing students understood issues related to periodontal diseases; however, the majority of participants were not confident about their understanding and recommended including more detailed oral health content in their nursing curriculum.

#### Varying attitudes of nursing students towards oral health care

Three studies conducted in the US [43], Japan [45], and Turkey [44] evaluated the attitudes of nursing students towards providing oral health care. Clemmens et al. [43] found that nursing students felt oral health care to be an essential component for effective nursing practice. A different trend was observed in nursing students from Turkey and Japan. Nursing students from Turkey often avoided going to a dentist until they developed a painful oral condition [44]. In Japan, the attitudes of nursing students toward oral health appeared negative, with 39.2% of students stating that they were not interested in learning about oral health and practice [45].

#### Need for further oral health care education for nursing students

Seven of the 11 studies provided suggestions for including an oral health component in nursing curricula. Six of the seven studies [46–48, 50–52] focussed on an interprofessional oral health education model. The remaining study [49] provided information about resources for older people’s oral health care for nursing curricula.

#### Available learning resources for nursing students to promote oral health

Lewis et al. [49] evaluated the relevance of “Building Better Oral Health Communities” (BBOHC) resources for students undertaking a Bachelor of Nursing, Diploma of Nursing, or Certificate III in Aged Care. The BBOHC resources were developed as a part of the Australian government-funded project for aged care workforce training in older people’s oral health care [13]. The BBOHC consists of five modules: 1) better oral health care, 2) dementia and oral care, 3) understanding the mouth, 4) care for natural teeth, and 5) care for dentures. Participating students were highly satisfied with the content of this resource [49]. Student learning outcomes showed consistently positive attitudes and substantial enhancements in oral health care knowledge and skills. Educators found the BBOHC content highly relevant in reinforcing a comprehensive approach to older people’s oral health care, which included learning about the consequences of poor health, dry mouth problems, oral health assessment, oral health planning, and timely referral. Educators also found the resources useful in building students’ skills in daily oral hygiene practice by increasing awareness about oral hygiene products, tooth brushing techniques, denture cleaning, and techniques to manage care resistive behaviours [49].

#### Value of an Interprofessional education model

An interprofessional education (IPE) model in which nursing students work with, learn from, and contribute to the oral-systemic knowledge of dental and other allied health students has been found effective in improving understanding of nursing students towards their role in oral health care [46–48, 50–52]. All the studies focussing on IPE were conducted in the US except the study by Grant et al. [46], which was conducted in Canada.

As a result of their IPE experiences in lectures and simulation exercises, nursing students showed significant improvement in oral health behaviour, knowledge, and attitudes regarding the importance of oral health care [47, 52]. Interprofessional education and practice experiences also increased nursing students’ confidence in conducting oral examinations and providing counselling [50]. IPE provided the platform for students to explore oral-systemic disease connections in a supportive team culture [46, 48, 51]. IPE helped nursing students to learn oral risk assessments, identify common oral pathologies, engage in oral hygiene activities, use fluoride varnish and work with students from other professions to promote oral health [47]. IPE clinical experiences focussing on oral-systemic health were valuable in enhancing shared professional skills with hands-on care, facilitating effective communication, and working as a team to develop an integrated plan of care to ensure holistic care [50, 51]. Nursing students’ experiences in interprofessional clinical practice were instrumental in understanding how underserved and rural communities could benefit from accessing multiple providers at one place on the same day [50].

## Discussion

### Summary of evidence

This review of 11 identified studies documented limited oral health knowledge and varying attitudes (both favourable and unfavourable) of nursing students towards oral health care. The review identified available learning resources and highlighted the importance of an interprofessional education and practice approach in improving oral health knowledge and attitudes among nursing students.

Growing evidence of the relationship between poor oral health and general systemic health requires urgent attention. The inclusion of oral health care education in nursing curricula, integrated with an interprofessional approach, will strengthen the capability and interest of future nurse practitioners to include evidence-based effective oral health care in routine nursing care. It is important to understand that “oral health care” can be interpreted differently by different health professionals [56]. For nursing practice, oral health care includes collaboration with dental, medical, and allied health professionals. For nursing students, this entails understanding the factors affecting people’s oral health and oral health-related quality of life, ensuring daily oral care practice, and being able to complete an oral health screening. Such screening includes checking the status and function of oral structures and dentures, swallowing ability, nutritional status, asking each person’s perspective about their oral and general health and whether they have any concerns, and making appropriate referrals. Daily oral care for older people in residential care includes assisting with evidence-based oral hygiene, use of saliva substitutes when appropriate, water hydration, desensitising agents, lip balms, denture cleaning tablets, pastes and adhesive pastes, and fluoride varnishes.

Older people are at particular risk for poor oral health. This review showed a significant gap in the current literature on nursing students’ knowledge of oral health care for older people and how this gap is best addressed through interprofessional education and practice. Interprofessional education and practice is one of the best ways to improve both nursing students’ awareness of the importance of oral health and their role in improving access to and providing oral health services [48, 57]. IPE facilitates collaborative work in varying health care settings including educational institutes [52], dental health clinics [47], mobile clinics in underserved areas [48] and hospitals [51]. While challenges remain in coordinating curricula across disciplines to facilitate students’ involvement in IPE initiatives, providing nursing students with opportunities to include oral health assessments in their assessment of overall body function would significantly improve the health outcomes of older people [32, 58]. IPE models have been implemented successfully in many graduate nursing programs in addition to undergraduate nursing programs [46, 59–63]. The “Smile for Life-National Oral Health Curriculum” has been popular with graduate nursing students [17, 59, 62–64]. This comprehensive oral health curriculum was initially developed in 2005 for primary health workers. It is freely available online and could be readily integrated into IPE activities in nursing curricula (www.smilesforlifeoralhealth.com) [65].

Implementing IPE into nursing curricula requires thought, time, and careful planning to ensure that students from other health-related programs can participate [46, 63, 66]. Organising IPE with students and faculty from different health programs with different health knowledge makes IPE challenging [48]. Flexibility, willingness, and cooperation among all professionals are needed for effective collaborative and interprofessional learning [46]. Establishing academic credit for students participating in IPE is an effective way to involve and encourage students in collaborative learning about oral health [46, 66].

Effective oral health education must include clinical practice and ideally interprofessional clinical practice. The best way to translate oral health learning to practice is to shift from the traditional physical assessment approach that is Head, Eyes, Ears, Nose, Throat (HEENT) to the Head, Eyes, Ears, Nose, Oral cavity, and Throat (HEENOT) approach for the assessment, diagnosis, and treatment of oral-systemic health [17]. The HEENOT approach ensures that one does “NOT” leave oral health assessment out of any medical history and physical examination. The success of the HEENOT approach was evidenced by more than 1000 referrals to the Nursing Faculty Practice (NFP) from New York University (NYU) dental clinics between 2008 and 2014. The HEENOT approach resulted in increased care appointments and more than 500 referrals to NYU dental clinics from the NFP [17]. Another collaborative model provided students in nursing, dental hygiene, and health services management with community-based experience providing affordable oral health services and oral health education [67]. Nursing students’ involvement in early detection of oral health issues and appropriate, timely referral to a dentist can ensure minimal cost treatment and improve patient-centred care [31].

This systematic review is a valuable initial step in identifying the current knowledge and attitudes of nursing students towards providing oral health care and recognising factors to reinforce their interest in oral health care, particularly for older people [45]. Results have several implications for nursing students, nursing educators, nursing education accreditation authorities, and researchers. Nursing students need to understand the importance of oral health, the relationship of poor oral health to systemic disease, the importance of their competency in oral health practices, and their important role in maintaining the health of older people. Oral health education and practical experience occur best in an interprofessional situation to build confidence, motivation, knowledge, and skills. Nursing educators need to understand and implement an interprofessional approach to oral health education and practice in nursing curricula. Nursing education accreditation authorities need to pay attention to develop guidelines to promote oral health care learning and practice among nursing students who are future health professionals. IPE improves workplace practices and productivity, patient health outcomes, staff morale, patient safety, and enables better access to health care [28]. Ongoing rigorous research is required to understand the extent to which oral health is addressed in nursing curricula in Australia and to evaluate the inclusion and impact of oral health content, delivered through interprofessional education and clinical practice, in undergraduate nursing curricula.

## Limitations

Most of the reviewed studies were conducted in the United States and had small sample sizes belonging to a single location; therefore, the results cannot be generalised. Some intervention studies had quasi-experimental designs, and there was a lack of blinding of the intervention leading to questions on the trustworthiness of the study results. Self-report questionnaires were used in the identified studies which may be biased by respondent beliefs. The long-term evaluation results of an integrated oral health learning model are still not available to check the effectiveness of IPE in building nursing students’ capacity in oral healthcare delivery.

## Conclusion

This review supports the need to integrate oral health education into nursing curricula, ideally through an IPE approach, to increase nursing students’ knowledge and ability to provide oral care, particularly to maintain the health of older people, and to interest students in providing effective oral care. There is a need to conduct rigorous well-designed studies about how best to achieve this and measure its success. A future nursing workforce with competence in oral health care will help to improve the oral health and quality of life of all people, especially those who are older and dependent on others for care.

## Data Availability

All data generated or analysed during this study is included in this article.

## Abbreviations

CINAHL: Cumulative Index to Nursing and Allied Health Literature
OECD: Organisation of Economic Co-operation and Development
IPE: Interprofessional education
MMAT: Mixed Methods Appraisal Tool
BBOHC: Building Better Oral Health Communities
HEENOT: Head, Eyes, Ears, Nose, Oral cavity, and Throat
NYU: New York University
